# Diversification of the Histone Acetyltransferase GCN5 through Alternative Splicing in *Brachypodium distachyon*

**DOI:** 10.3389/fpls.2017.02176

**Published:** 2017-12-21

**Authors:** Alexandre Martel, Hardev Brar, Boris F. Mayer, Jean-Benoit Charron

**Affiliations:** Department of Plant Science, McGill University, Sainte-Anne-de-Bellevue, QC, Canada

**Keywords:** alternative splicing, histone acetylation, GCN5, SAGA, *Brachypodium distachyon*, functional diversification

## Abstract

The epigenetic modulatory SAGA complex is involved in various developmental and stress responsive pathways in plants. Alternative transcripts of the SAGA complex's enzymatic subunit GCN5 have been identified in *Brachypodium distachyon*. These splice variants differ based on the presence and integrity of their conserved domain sequences: the histone acetyltransferase domain, responsible for catalytic activity, and the bromodomain, involved in acetyl-lysine binding and genomic loci targeting. *GCN5* is the wild-type transcript, while alternative splice sites result in the following transcriptional variants: *L-GCN5*, which is missing the bromodomain and *S-GCN5*, which lacks the bromodomain as well as certain motifs of the histone acetyltransferase domain. Absolute mRNA quantification revealed that, across eight *B. distachyon* accessions, *GCN5* was the dominant transcript isoform, accounting for up to 90% of the entire transcript pool, followed by *L-GCN5* and *S-GCN5*. A cycloheximide treatment further revealed that the *S-GCN5* splice variant was degraded through the nonsense-mediated decay pathway. All alternative *BdGCN5* transcripts displayed similar transcript profiles, being induced during early exposure to heat and displaying higher levels of accumulation in the crown, compared to aerial tissues. All predicted protein isoforms localize to the nucleus, which lends weight to their purported epigenetic functions. S-GCN5 was incapable of forming an *in vivo* protein interaction with ADA2, the transcriptional adaptor that links the histone acetyltransferase subunit to the SAGA complex, while both GCN5 and L-GCN5 interacted with ADA2, which suggests that a complete histone acetyltransferase domain is required for BdGCN5-BdADA2 interaction *in vivo*. Thus, there has been a diversification in *BdGCN5* through alternative splicing that has resulted in differences in conserved domain composition, transcript fate and *in vivo* protein interaction partners. Furthermore, our results suggest that *B. distachyon* may harbor compositionally distinct SAGA-like complexes that differ based on their histone acetyltransferase subunit.

## Introduction

The acetylation of lysine residues on histone tails is an epigenetic mark that is involved in regulating important plant processes such as flowering time, flower development and abiotic stress responses (Bertrand et al., 2003; He et al., 2003; Cohen et al., 2009; Moraga and Aquea, 2015). GCN5 is a conserved histone acetyltransferase that acts as one of the enzymatic subunits of the multi-protein SAGA complex, and has been shown to associate with up to 40% of *Arabidopsis thaliana* gene promoters (Benhamed et al., 2008; Koutelou et al., 2010). It is known to acetylate histones present at the promoter of genes, an epigenetic mark that is correlated with increased transcription rates (Sterner and Berger, 2000; Robert et al., 2004; Rosaleny et al., 2007). GCN5's enzymatic capability is conferred by a HAT domain, which may be subdivided into four motifs, arranged as C, D, A, and B (Dyda et al., 2000; Sterner and Berger, 2000). Acetyl-CoA provides the acetyl group required for GCN5's enzymatic function, and the binding of this coenzyme occurs in the cleft delineated by motifs A and B (Dyda et al., 2000; Sterner and Berger, 2000). Thus, the motifs that makeup the GCN5 HAT domain are responsible for its ability to influence gene expression.

The acetylation of histone H3 and H4 tails by GCN5 occurs on multiple lysine residues in a stepwise fashion, H3K14 being the primary site of acetylation (Kuo et al., 1996; Grant et al., 1997; Benhamed et al., 2006; Earley et al., 2007; Kuo and Andrews, 2013; Cieniewicz et al., 2014; Mahrez et al., 2016). The bromodomain is GCN5's second conserved protein domain, responsible for recognizing and binding acetylated lysine residues, and is required for proper GCN5-dependent deposition order (Hassan et al., 2007; Zeng et al., 2008; Cieniewicz et al., 2014). The bromodomain is also involved in promoter binding and targeting by GCN5, and increases the acetylation of H3 tails when the other H3 in the same nucleosome is acetylated (Hassan et al., 2002; Benhamed et al., 2008). Therefore, the bromodomain may influence the targeting and extent of GCN5's enzymatic activity.

Across all the organisms in which GCN5 has been characterized, it is physically linked to the remainder of the SAGA complex through an interaction with the transcriptional adaptor ADA2 (Candau and Berger, 1996; Candau et al., 1996, 1997; Stockinger et al., 2001; Bhat et al., 2003; Fan et al., 2004; Mao et al., 2006; Gamper et al., 2009). The region required for the GCN5-ADA2 interaction differs among organisms. In yeast, this interaction requires the region present between the HAT and bromodomain, while in *A. thaliana*, which contains two copies of ADA2, the required region of interaction is exclusively the HAT domain (Candau and Berger, 1996; Candau et al., 1997; Mao et al., 2006). Such an example displays the differences in the composition of the SAGA complex across different organisms. However, in all known instances, GCN5 interacts with one or more ADA2 protein(s) in order to link it to the remainder of the SAGA complex.

In addition to its role as the physical link between GCN5 and the SAGA complex, ADA2 increases GCN5's substrate specificity to include histones in a nucleosomal context, compared to free histones only, when GCN5 is alone (Grant et al., 1997, 1999; Sterner and Berger, 2000; Gamper et al., 2009). Furthermore, ADA2 allows for GCN5 to be recruited to genomic loci indirectly through interactions that occur between other SAGA complex members and transcription factors, which complement the direct transcription factor interactions GCN5 exhibits (Belotserkovskaya et al., 2000; Kuo et al., 2000; Brown et al., 2001; Lang et al., 2001; Stockinger et al., 2001; Barbaric et al., 2003; Bhat et al., 2004; Gao et al., 2007; Nagy and Tora, 2007; Hirsch et al., 2015; Setiaputra et al., 2015). SGF29, another SAGA complex member, recognizes H3K4me2/3 and increases SAGA recruitment and GCN5-dependent histone acetylation at sites bearing such epigenetic marks (Bian et al., 2011; Schram et al., 2013; Ringel et al., 2015). Thus, association with the SAGA complex expands GCN5's function.

Alternative splicing is a widespread process that may increase the functional diversity of genes; in *A. thaliana* an estimated 61% of multi-exon containing genes may undergo alternative splicing (Marquez et al., 2012). This cellular process is known to functionally regulate certain epigenetic modulators in plants (Shen et al., 2016). Of further interest, a *GCN5* splice variant has been identified in Humans, which includes an N-terminal extension to its sequence that represents a PCAF domain (Smith et al., 1998). Interestingly, the only known *GCN5* transcript present in *Drosophila melanogaster* is homologous to the entire region of the longer human *GCN5* splice variant, containing the PCAF homology domain in addition to the HAT and bromodomain sequences (Smith et al., 1998). This additional PCAF domain is believed to be responsible for the capability to interact with the CBP and p300 transcriptional coactivators (Yang et al., 1996; Smith et al., 1998). Such examples of alternative splicing allows for a single gene to generate functionally distinct protein products. However, this is not always the case, as certain splice variants are actively degraded through a ribosomal-dependent cellular process termed nonsense-mediated decay (NMD) (Lareau et al., 2007; Saltzman et al., 2008; Kwon et al., 2014). Therefore, alternative splicing is known to influence the functional diversity of epigenetic modulators.

This study describes the functional characterization of *B. distachyon GCN5*, as well as of two novel *GCN5* splice variants, which differ from the former based on the presence and integrity of conserved protein domains.

## Materials and methods

### Plant material

Unless otherwise noted, *B. distachyon* accession Bd21 was used for all experiments. Seeds were imbibed for 4 h and subsequently surface sterilized by first removing the lemma, treating for 30 s with 70% ethanol, washing twice in sterile water, treating for 3 min with 1.3% bleach and washing three times in sterile water prior to stratification in the dark at 4°C for 5–7 days. Plants were grown at 22°C in an E15 Conviron growth cabinet with a 16/8 h light/dark photoperiod and 130 μmol/ms of light.

For *A. thaliana*, ecotype Col-0 was used for all experiments. Seeds were sterilized by inverting in 70% ethanol for 30 s, 1.3% bleach for 5 min and rinsed four times with sterile water. Sterile seeds were stratified at 4°C for 2–4 days in the dark prior to planting.

### Sequence analysis of *BdGCN5* alternative transcripts

Nucleic acid sequences of *GCN5* (XM_003573876.3), *S-GCN5* (MG552853), and *L-GCN5* (MG552854) were aligned using the Clustal Omega multiple sequence alignment tool (Sievers et al., 2011).

### Gene expression analysis

#### Absolute RNA quantification

Plasmid DNA was extracted from *Escherichia coli* strain DH5-α carrying pGEM®-T Easy (Promega) constructs containing *GCN5, S-GCN5*, or *L-GCN5* using the EZ-10 Spin Column Plasmid DNA Minipreps Kit (Bio Basic Inc.). DNA concentration was quantified using a ND-1000 spectrophotometer (NanoDrop). A dilution series was prepared in order to generate a standard curve ranging in concentration from 10^−13^ to 10^−20^ mol/μl.

Aerial tissues from 2-week-old Bd21, Bd3-1, Bd18-1, Bd2-3, Bd21-3, Bd1-1, Bd30-1, and Bd29-1 *B. distachyon* individuals were collected and frozen in liquid nitrogen. RNA extractions were performed using EZ-10 Spin Column Plant RNA Mini-Preps Kit (Bio Basic Inc.). cDNA synthesis was performed using iScript Advanced cDNA Synthesis Kit (BioRad Laboratories Inc.). qPCR was performed using Green-2-Go qPCR Mastermix (Bio Basic Inc.) and gene-specific primers in a CFX Connect™ (BioRad Laboratories Inc.). All genes were normalized to both *SamDC* and *Ef1-*α (Hong et al., 2008). Primers used in this study are listed in Supplementary Table 1. Statistical analyses were performed using the JMP software. Three biological replicates were used for the absolute quantification as well as for all other gene expression analyses.

#### Nonsense-mediated decay assays

Aerial tissues of 2 week-old *B. distachyon* accession Bd21 or 2 week-old *A. thaliana* ecotype Col-0 were treated by adding them to 1X MS in 15 ml polypropylene tubes (Sarstedt Inc.) supplemented with 0.005% Silwet-77, and either cycloheximide (Sigma-Aldrich) to a final concentration of 20 μM dissolved in DMSO, or DMSO only (mock). Polypropylene tubes were continually inverted for 4 h prior to sampling and freezing in liquid nitrogen. RNA extractions and qPCR were performed as described above.

Collection of *B. distachyon* tissues for *BdGCN5* spatial expression analysis: Bd21 plants were grown in soil and aerial or crown tissues were collected at 3 weeks of age and immediately frozen in liquid nitrogen. RNA extractions and qPCR were performed as described above.

#### Abiotic stress treatments

All treatments were performed in sterile media (1X MS, 0.9% agar), with 2-week-old plants grown in culture tubes. Heat exposure: plants were transferred to 42°C and samples were collected at 30 min, 1 and 4 h post-treatment. Cold exposure: plants were transferred to 4°C and samples were collected at 1, 2, 4, 6, 8, 10, 12, 24 h post-treatment. Untreated plants grown at 22°C were concurrently collected as controls. All replicates of abiotic stress treatments were applied at the same time of day.

#### Primer design

**G**ene-specific primers were designed using NCBI's Primer-Blast software and ordered from Integrated DNA Technologies Inc.

### Subcellular localization

*GCN5, S-GCN5*, and *L-GCN5* coding sequences were cloned into the BglII sites of the pAVA321 localization vector in *E. coli* DH5-α, resulting in an N-terminal GFP tag (von Arnim et al., 1998). All plasmid constructs were validated by Sanger sequencing. Plasmid DNA was isolated for each of the following pAVA321 constructs *GCN5, S-GCN5, L-GCN5*, and empty vector using the EZ-500 Spin Column Plasmid DNA Maxipreps Kit (Bio Basic Inc.). Isolated DNA was diluted to a concentration of 3 ng/μl and used for biolistic particle bombardment as described below.

M-17 tungsten microcarriers (BioRad Laboratories Inc.) were prepared by suspending 100 mg in 1 ml of 100% ethanol. Microcarriers were centrifuged at 14,000 rpm for 5 min and re-suspended in 1 ml of 100% ethanol. This washing procedure was repeated 5 times. Following the final wash step, the microcarriers were re-suspended in 1 ml of 100% ethanol and stored at −20°C. Prior to use, prepared microcarriers were sonicated at 4°C for 1 min at the maximum setting using a Bioruptor UCD-200 (Diagenode). To adsorb DNA, 2 μl of plasmid DNA was mixed with 2 μl of 100% ethanol and vortexed for 10 s. Eight microliters of 100% ethanol was added to the above mixture and vortexed for 10 s. Twenty microliters of prepared tungsten microcarriers were added to the above mixture and vortexed for 15 s. Eight microliters of the DNA-adsorbed microcarriers were loaded onto Swinnex filter holders (Merck Millipore) and allowed to dry for 2 min.

Onion epidermal peels were placed on 1X MS, 1.5% agar plates and bombarded using a particle inflow gun (Vain et al., 1993). The solenoid timer was set to 50 ms and the helium pressure to 85 psi. Following bombardment, onion samples were placed in the dark at 28°C for 16–24 h then visualized using a V20 Discovery Stereomicroscope (Zeiss) equipped with an X-Cite Series 120Q UV lamp (Lumen Dynamics Group Inc.).

### Bimolecular fluorescence complementation

The pDOE set of vectors was used for this BiFC assay (Gookin and Assmann, 2014). The coding sequence of *B. distachyon ADA2* was cloned into the BamHI-SpeI sites of MCS1, while the coding sequences of *GCN5, S-GCN5*, and *L-GCN5* were cloned into the AatII-RsrII sites of MCS3 and maintained in *E. coli* DH5-α. All plasmid constructs were validated by Sanger sequencing.

Plasmid DNA from positive clones were extracted using the EZ-10 Spin Column Plasmid DNA Minipreps Kit (Bio Basic Inc.) and electroporated into competent *Agrobacterium tumefaciens* strain GV3101::pMP90 using an Eppedorf Multiporator® and the suggested parameters for *A. tumefaciens* (Eppendorf Multiporator® Protocol No. 4308 915.502−12/2001). Positive *A. tumefaciens* were confirmed by PCR at both the MCS1 and MCS3 sites.

To perform the infiltration, confirmed *A. tumefaciens* clones were grown overnight at 28°C in LB media, supplemented with 30 μg/ml gentamycin and 50 μg/ml kanamycin. The following day, cultures were supplemented with 150 μM of acetosyringone and incubated for 2 h. Subsequently, cells were pelleted and resuspended in infiltration solution (10 mM MES, 10 mM MgCl_2_, and 150 μM acetosyringone) to a final OD600 of 0.7. This solution was infiltrated into the abaxial surface of *Nicotiana benthaminana* leaves and the plants were grown in the dark for 48 h. Two hours prior to visualization, *N. benthamiana* leaves were infiltrated with a solution of 1 mg/ml DAPI, 0.5% Triton X-100, 10 mM MES, and 10 mM MgCl_2_. Leaf epidermal cells were visualized using a AxioImager.Z1 (Zeiss).

## Results

### *B. distachyon* has at least three distinct *GCN5* alternative transcripts

**Figure 1:**
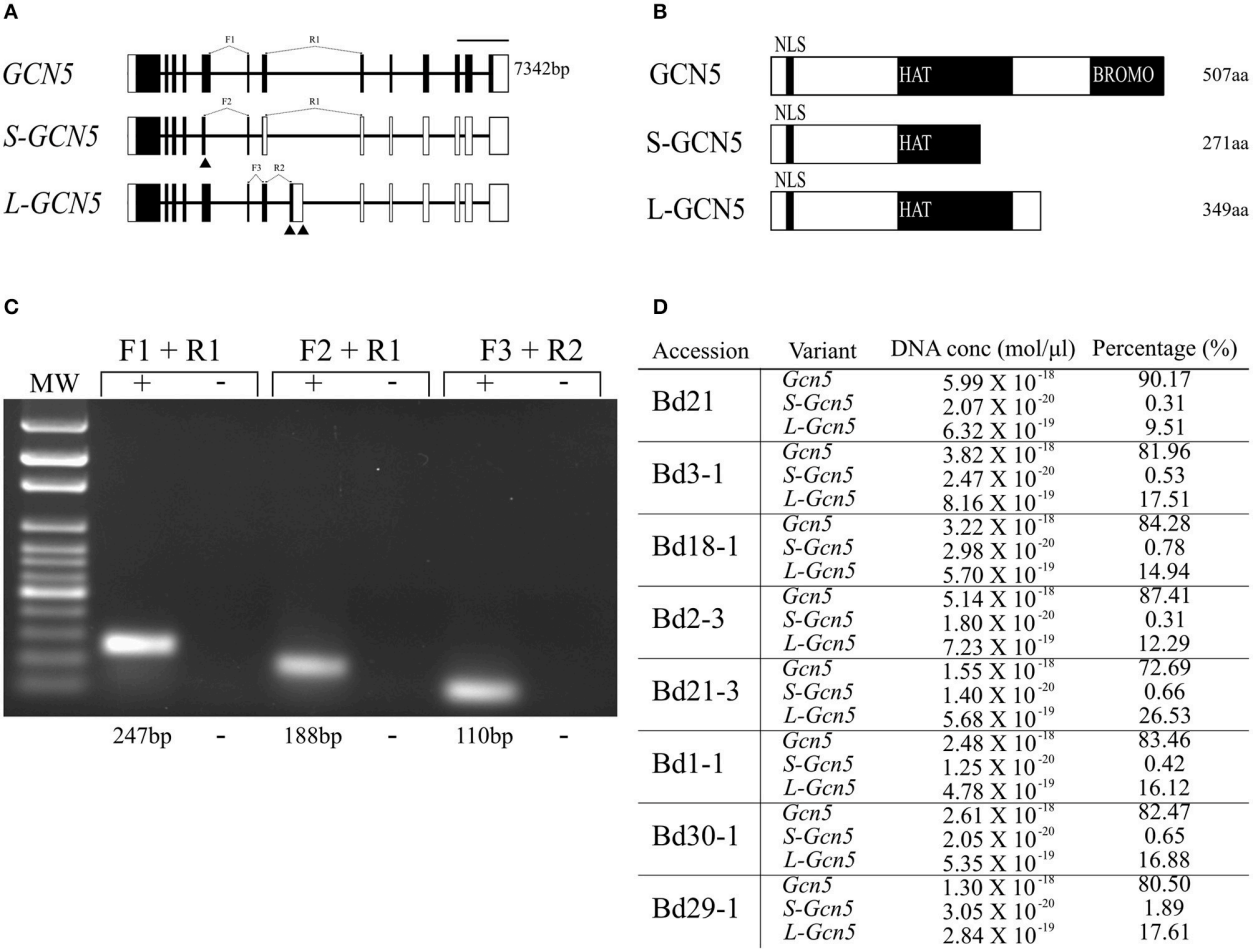
*BdGCN5* splice variants differ in their conserved domain composition and absolute transcript abundance. **(A)** Genomic representation of *GCN5* alternative transcripts. Exons are depicted as black boxes, UTRs are depicted as white boxes. Forward (F) and reverse (R) primers used for qPCR are presented at their appropriate locations. Black arrows represent alternative splice sites. Scale bar = 1,000 bps. **(B)** Predicted protein product representation of BdGCN5 variants. Black boxes represent conserved protein domains. NLS, nuclear localization signal; HAT, histone acetyltransferase domain; and BROMO, Bromodomain. **(C)** Confirmation of specific detection of *BdGCN5* splice variants through qPCR, using the primer pairs depicted in **(A)**, MW, molecular weight marker. **(D)** Absolute RNA quantification of each *BdGCN5* variant is presented as an absolute value in mol/μl of input cDNA (the cDNA reaction contained 75 ng/μl of input RNA from aerial tissue extractions), as well as the percentage of the total *BdGCN5* transcript pool in each tested accession.

Efforts to clone the *GCN5* transcript from *B. distachyon* accession Bd21 led to the identification of two previously unknown splice variants. The first alternative transcript, termed *S-GCN5* due to its coding region being the shorter of the two splice variants, results from an alternative splice site that shortens exon 5 (Figure 1A). The second, termed *L-GCN5* due to its coding region being the longer of the two splice variants, results from two alternative splice sites that include a supplementary exon in the transcript, between the locations of exons 7 and 8 in the *GCN5* transcript (Figure 1A). Both *S-GCN5* and *L-GCN5* result in earlier stop codons when compared to *GCN5*, reducing the length of the coding region and expected protein products (Figure 1A).

Multiple sequence alignments and analyses of the predicted domains of the protein isoforms demonstrated that the alternative splicing events altered the conserved domain composition of the variants, when compared to GCN5 (Figure 1B, Supplementary Figure 1). The protein sequence of both splice variants was missing the bromodomain, however the L-GCN5 protein retained an integral HAT domain, while the S-GCN5 protein only retained a part of the HAT domain (Figure 1B). More specifically, of the four motifs that make up the HAT domain, the S-GCN5 protein sequence contained the complete C and D motifs, and part of the A motif, while the B motif was missing (Supplementary Figure 1). Therefore, at least three distinct *BdGCN5* alternative transcripts are present in *B. distachyon*, which differ based on the composition and integrity of their conserved protein domains.

### *GCN5* is the dominant transcript isoform

Absolute mRNA quantification was performed to gain insight regarding the abundance of each *BdGCN5* alternative transcript in aerial tissues of eight *B. distachyon* accessions (Bd21, Bd3-1, Bd18-1, Bd2-3, Bd21-3, Bd1-1, Bd30-1, and Bd29-1) known to display natural diversity in many phenotypic characteristics as well as photoperiod and vernalization requirements (Colton-Gagnon et al., 2014; Ream et al., 2014; Tyler et al., 2014). Variant-specific primers were validated and standard curves were generated in order to quantify absolute RNA levels (Figure 1C, Supplementary Figure 2). *GCN5* was the dominant transcript, accounting for 73–90% of the entire *BdGCN5* transcript pool, followed by *L-GCN5* at 9–26% and *S-GCN5* at <2% (Figure 1D). Across all accessions, Bd21 had the highest proportion of *GCN5* RNA as well as the lowest of both *S-GCN5* and *L-GCN5*, while Bd21-3 had the highest proportion of *L-GCN5* and Bd29-1 of *S-GCN5*. Thus, the quantity that each alternative *BdGCN5* transcript accounts for within aerial tissues was variable among accessions of *B. distachyon* grown under control conditions. However, *GCN5* was always dominant, followed by *L-GCN5* then *S-GCN5*.

### *S-GCN5* is degraded through the nonsense-mediated decay pathway

Alternative splicing that results in transcript variants with early stop codons and long 3′ UTRs may be targets of nonsense-mediated decay (NMD), a ribosome-dependent, splice variant degradation pathway that is involved in actively suppressing transcript abundance levels. Both *S-GCN5* and *L-GCN5* have such sequence features and were therefore selected for NMD analysis. Since the NMD pathway has yet to be investigated in *B. distachyon*, sequence homology analysis of known NMD-requiring components from *A. thaliana* was performed in *B. distachyon* sequence databases, in order to confirm that this organism contains the appropriate machinery. *B. distachyon* encodes homologs of all currently known NMD-requiring proteins characterized in *A. thaliana*, with protein sequence similarities ranging from 56 to 82% (Supplementary Figure 3).

**Figure 2:**
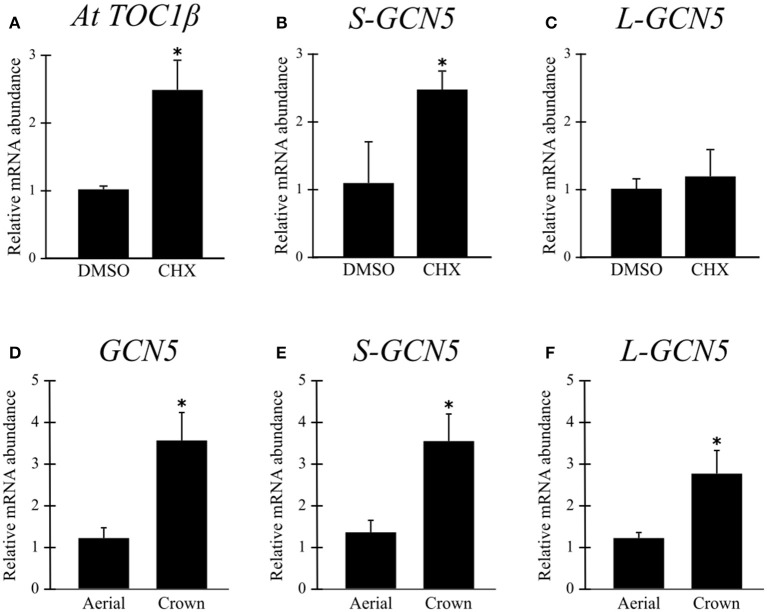
The *S-GCN5* transcript is degraded by the nonsense-mediated decay pathway. **(A–C)** Relative transcript abundance of *AtTOC1*β (positive control), *S-GCN5*, and *L-GCN5* following cycloheximide (CHX) or control (DMSO) treatments. **(D–F)** Relative transcript abundance of *GCN5, S-GCN5*, and *L-GCN5* in aerial and crown tissues. Error bars represent the standard deviation of three biological replicates. An asterisk indicates a statistically significant difference (*P* < 0.05).

A cycloheximide treatment inhibits the ribosome, and thus also inhibits the NMD pathway. Analysis of cycloheximide-treated *B. distachyon* aerial tissues indicated that the *S-GCN5* transcript is actively degraded by NMD under normal conditions, while the *L-GCN5* transcript is not thusly affected (Figures 2B,C). As there are no currently known NMD targets in *B. distachyon*, a positive control for the effectiveness of the treatment was performed on a previously identified NMD target in *A. thaliana*, which confirmed the effectiveness of the assay (Figure 2A).

### *BdGCN5* variants are differentially expressed in different *B. distachyon* tissues and following exposure to various abiotic stresses

**Figure 3:**
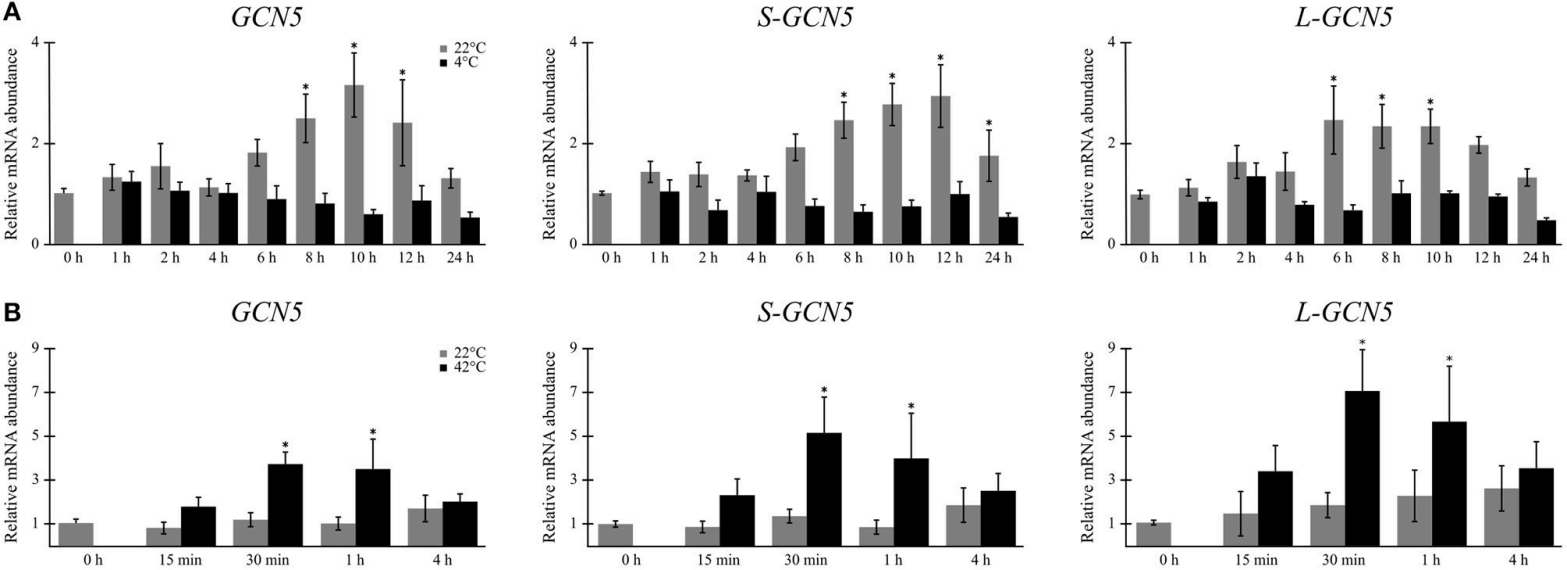
*BdGCN5* alternative transcripts are differentially expressed following exposure to temperature extremes. Relative expression profiles of each *BdGCN5* alternative transcript in aerial tissues following exposure to: **(A)** cold (4°C) or **(B)** heat (42°C) for the indicated amounts of time. Error bars represent the standard deviation of at least three biological replicates. An asterisk indicates a statistically significant difference (*P* < 0.05).

Relative expression data indicated that mRNA abundance levels of all the alternative *BdGCN5* transcripts are significantly higher in crown tissue, when compared to aerial tissue (Figures 2D–F). Exposure of *B. distachyon* individuals to low (4°C) temperatures demonstrated that the apparent circadian variation in relative transcript abundance of all *BdGCN5* variants is repressed, mainly between 6 and 12 h following cold treatment (Figure 3A). In contrast, exposure to high (42°C) temperatures indicated that the abundance of all alternative transcripts was significantly increased following 30 min and 1 h of heat exposure and returns to control levels after 4 h of the applied heat treatment (Figure 3B). All *BdGCN5* transcripts followed similar trends across tested treatments and tissues.

### All BdGCN5 isoforms localize to the nucleus

**Figure 4:**
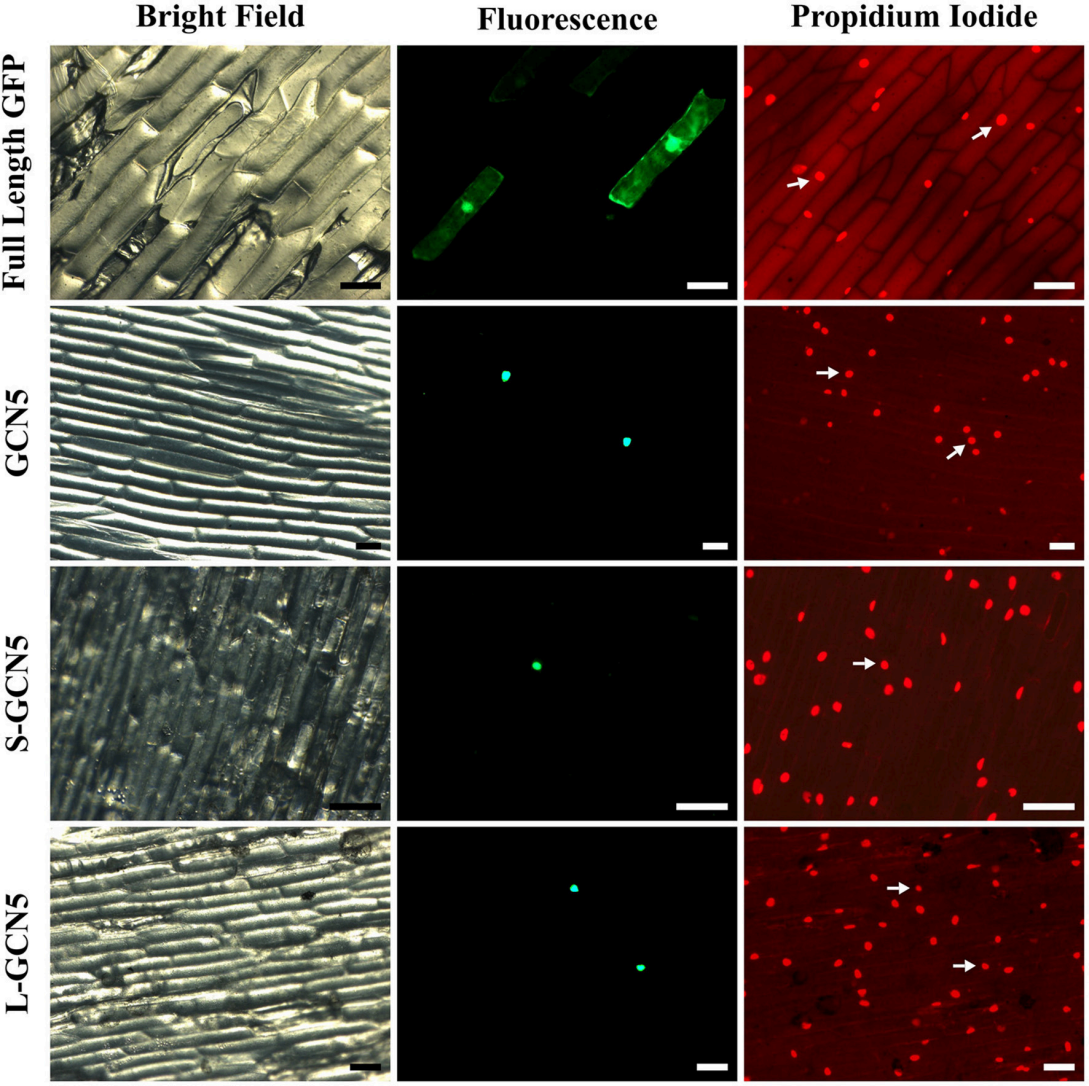
All BdGCN5 isoforms localize to the nucleus. GFP was N-terminally tagged to *GCN5, S-GCN5*, and *L-GCN5* using the pAVA321 localization vector. Untagged GFP, as well as the N-terminally GFP-tagged constructs, were used for particle bombardment of onion scale cells. Images of bright field, fluorescence (GFP filter) and following propidium iodide staining are presented. Arrows indicate nuclei detected under both propidium iodide and GFP filters. Scale bar = 100 μm.

To perform their function, histone acetyltransferase enzymes and other epigenetic modulators must localize to the nucleus. All *BdGCN5* transcript variants retain a single nuclear localization signal (Figure 1B, Supplementary Figure 1B). A localization assay using a N-terminal GFP tag in onion cells was performed to determine the *in vivo* localization of the predicted protein products. Note that NMD did not affect recombinant protein accumulation as exclusively the coding region was used to generate the constructs. All BdGCN5 variants' predicted protein products localized to the nucleus (Figure 4).

### GCN5 isoforms differ in their ability to interact with the SAGA complex member ADA2

**Figure 5:**
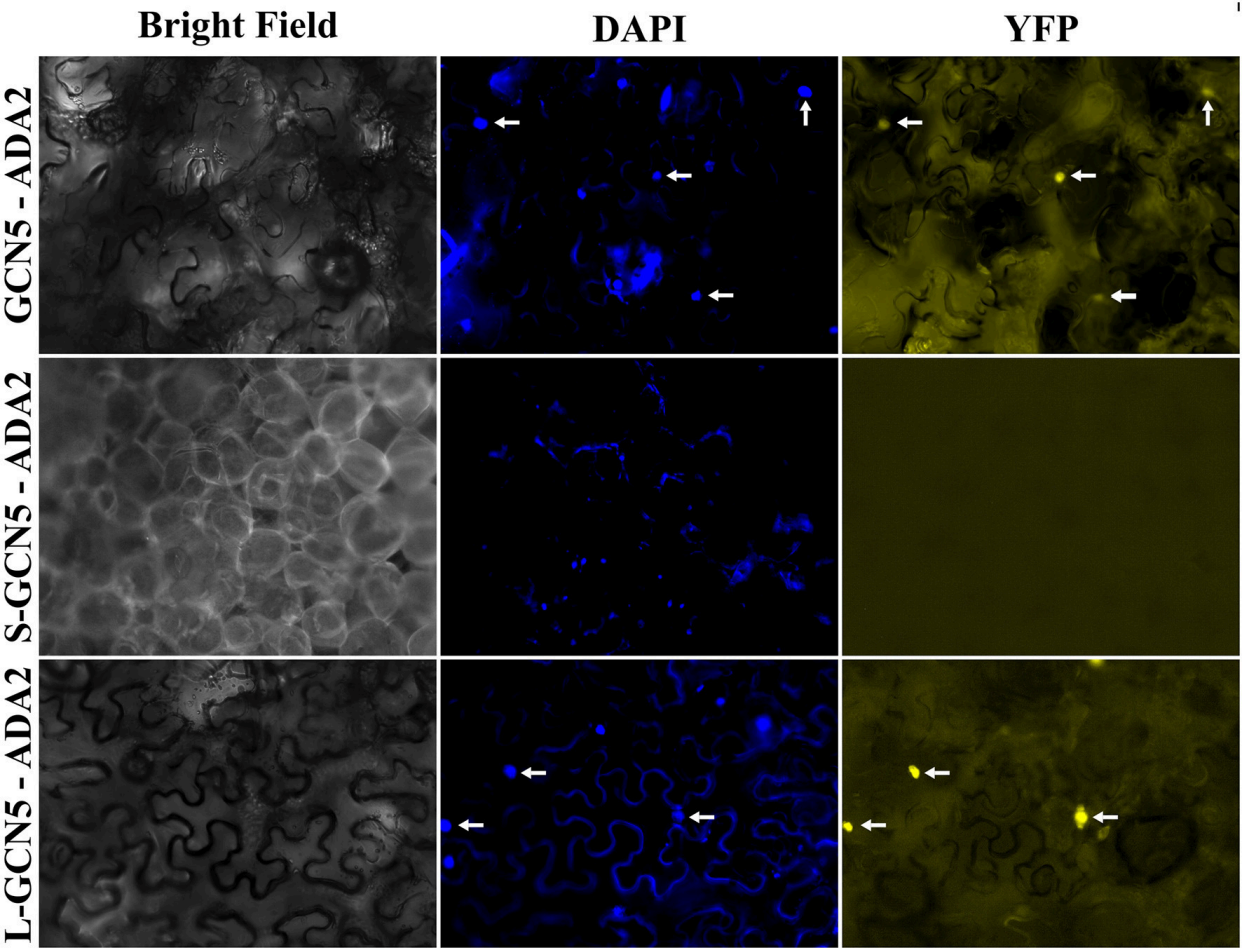
GCN5 and L-GCN5 interact with ADA2 *in vivo*. Representative images of *N. benthamiana* epidermal cells infiltrated with *A. tumefaciens* harboring the pDOE-8 bimolecular fluorescence complementation vector that contained either GCN5, S-GCN5 or L-GCN5, and ADA2. Images of bright field and fluorescence (YFP filter) and following DAPI staining are presented. Arrows indicate nuclei detected under both DAPI and YFP filters.

In all organisms in which GCN5 has been characterized, it has always been in the context of multi-protein complexes (Candau and Berger, 1996; Candau et al., 1996, 1997; Stockinger et al., 2001; Bhat et al., 2003; Fan et al., 2004; Mao et al., 2006; Gamper et al., 2009). Therefore, the ability for BdGCN5 variants to interact with ADA2, a transcriptional adaptor that physically links GCN5 to the remainder of the SAGA complex in other organisms, was assessed through a bimolecular fluorescence complementation experiment using the pDOE set of vectors (Gookin and Assmann, 2014). Infiltration into *N. benthamiana* demonstrated that exclusively GCN5 and L-GCN5 interacted with ADA2 *in vivo*, while S-GCN5 was incapable of such an interaction (Figure 5). Of note, the main difference between the L-GCN5 and S-GCN5 amino acid sequences is the presence of a complete HAT domain in L-GCN5, whereas S-GCN5's HAT domain is truncated (Figure 1, Supplementary Figure 1B). This information, coupled to the protein interaction results, indicated that a complete HAT domain was required for BdGCN5-ADA2 interaction in *B. distachyon*. This experiment was repeated with similar results with other pDOE vector constructs that tag the split-YFP sections in differing orientations. No fluorescence was observed when the appropriate controls were tested. The observed GCN5/L-GCN5—ADA2 interaction occurred in the nucleus, which further confirms the subcellular localization of the BdGCN5 protein variants determined in Figure 4. Therefore, GCN5 and L-GCN5 were capable of multi-protein complex formation through direct protein interaction with ADA2, and this interaction required a complete HAT domain.

## Discussion

Alterative splicing of a histone demethylase was recently demonstrated to occur in *Medicago truncatula*, where alternative splice sites result in early stop codons and loss of predicted amino acid sequence length (Shen et al., 2016). While functional differences were not investigated in this study, a subset of the alternative isoforms are favored following cold exposure, suggestive of a role in low temperature responses (Shen et al., 2016). Additionally, alternative splicing in *GCN5* has previously been observed in humans, where an isoform representative of the *B. distchyon GCN5* sequence exists, as well as an isoform that contains an additional N-terminal extension that is homologous to a region of the human PCAF protein, another histone acetyltransferase (Smith et al., 1998). This additional domain allows for interaction with CBP or p300, two transcriptional co-activators (Yang et al., 1996). The only known *GCN5* in *D. melanogaster* is homologous to this longer human *GCN5* sequence, as it contains the N-terminal region of PCAF homology (Smith et al., 1998). Previous cases of alternative splicing in epigenetic modifiers have thus already been demonstrated. Here, alternative *GCN5* transcripts identified in *B. distachyon* are presented. The splicing in these variants show similarities with the histone demethylase identified in *M. truncatula*, as they result in early stop codons and the loss of conserved protein domains (Figure 1A,B, Supplementary Figure 1B), as opposed to the previously identified *GCN5* variants in human that result in the gain of an additional domain.

The three transcriptional *BdGCN5* variants all retain an identical N-terminal region (amino acids 1–247; Figure 1B, Supplementary Figure 1). This region contains the only NLS present in the sequences and the nuclear localization of all BdGCN5 isoforms was determined using a GFP-tagging assay in onion cells (Figure 4). This suggests that, regardless of the functional differences among BdGCN5 variants, all isoforms that accumulate to significant levels in *B. distachyon* perform a cellular function in the context of a nuclear environment, characteristic of epigenetic modifiers.

The region responsible for the enzymatic capability of the BdGCN5 proteins is the HAT domain and is present in its entirety in both the *GCN5* and *L-GCN5* sequences, but is truncated in the *S-GCN5* variant. HAT domains may be further subdivided into four distinct motifs, organized in the following order: C, D, A, and B (Supplementary Figure 1B; Tercero et al., 1992; Neuwald and Landsman, 1997; Dutnall et al., 1998; Wolf et al., 1998; Dyda et al., 2000). The A and B motifs display the highest level of sequence conservation throughout acetyltransferase enzymes, while the C motif displays the lowest, and is dispensable for activity in certain histone acetyltransferases (Dyda et al., 2000; Tyler et al., 2006). This high level of conservation in motifs A and B is most likely due to their essential function in forming an acetyl-CoA binding site and the presence of known catalytic residues (Dyda et al., 2000; Toleman et al., 2004). The motifs missing in the *S-GCN5* sequence encompass the entirety of the B motif and a significant section of the A motif. The *S-GCN5* protein product is therefore missing regions involved in the binding of an essential catalytic substrate and containing key catalytic residues. This result is not unprecedented, as many other enzymes have alternatively spliced isoforms that lose catalytic function (Kelemen et al., 2013).

The final domain in the *BdGCN5* sequences is the bromodomain, which is only present in the *GCN5* sequence (Figure 1B, Supplementary Figure 1B). While the bromodomain is not enzymatically active, it may influence the protein's enzymatic capability as its function is to recognize and bind to acetylated lysine residues (Ornaghi et al., 1999; Owen et al., 2000; Sterner and Berger, 2000; Yang, 2004; Hassan et al., 2007; Cieniewicz et al., 2014). Furthermore, promoter binding of the GCN5 protein characterized in other organisms is partially dependent on the presence of an intact bromodomain (Hassan et al., 2002; Benhamed et al., 2008). In *A. thaliana*, truncation of GCN5's bromodomain resulted in loss of association with 11% of the promoters tested (Benhamed et al., 2008). Moreover, yeast GCN5 proteins lacking bromodomain function displayed increased initial acetylation rates and an altered order of lysine acetylation on histone tails (Cieniewicz et al., 2014). Therefore, the lack of a bromodomain in both of the *BdGCN5* splice variants may indicate a diversification of this histone acetyltransferase's function in terms of catalytic activity as well as histone lysine substrate preference.

Absolute RNA quantification of the *BdGCN5* alternative transcripts indicate that, across all accessions analyzed, *GCN5* is the dominant isoform, followed by *L-GCN5*, while *S-GCN5* contributes little to the total *BdGCN5* transcript pool. Additionally, the *B. distachyon* accessions selected for absolute transcript analysis are known to display phenotypic and stress responsive variability, processes that involve epigenetic mechanisms (He, 2009; Colton-Gagnon et al., 2014; Ream et al., 2014; Tyler et al., 2014; Mayer et al., 2015). While no direct link is hereby suggested, it is interesting to note that there is observable variation in the absolute accumulation of *BdGCN5* transcriptional variants across *B. distachyon* accessions.

The low absolute transcript values of the *L-GCN5* and *S-GCN5* splice variants are likely due to favoring of the splicing machinery for the *GCN5* isoform. Another contributing factor may be the nonsense-mediated decay pathway, which is known to degrade a subset of alternatively spliced transcripts (Lareau et al., 2007; Saltzman et al., 2008; Kwon et al., 2014). The NMD mechanism is conserved across many eukaryotic organisms and targets certain splice variants with early stop codons and long 3′ UTRs, characteristics that both of the *BdGCN5* splice variants share (Figure 1A; Conti and Izaurralde, 2005; Drechsel et al., 2013). Cycloheximide assays, which inhibit the ribosome's function and thus NMD, determined that *S-GCN5* transcript levels are indeed under the repression of the NMD pathway, while the *L-GCN5* transcript is not (Figures 2B,C).

In terms of relative transcript abundance, all *BdGCN5* variants follow similar trends, be it across tissues or following exposure to temperature extremes (Figures 2D–F, 3). Therefore, for the tested tissues and conditions, there is no favoring of the splicing machinery for one variant, as has been observed with other alternatively spliced genes in plants (Filichkin et al., 2010; Shen et al., 2016). The higher observed relative transcript abundance of all *BdGCN5* variants in crown tissue, when compared to aerial tissues, suggests a putative role in meristem-dependent processes (Figures 2D–F). This observation is not surprising as GCN5 is known to be involved in developmental mechanisms in other plant species (Bertrand et al., 2003; Vlachonasios et al., 2003; Cohen et al., 2009; Kornet and Scheres, 2009; Poulios and Vlachonasios, 2016; Chen et al., 2017). Following exposure to low temperatures, the apparent circadian increase in gene expression is repressed in all *BdGCN5* alternative transcripts (Figure 3A). Exposure of plants to cold is known to result in such a profile for certain transcripts that are not cold-inducible (Bieniawska et al., 2008). While relative transcript accumulation levels of the *BdGCN5* isoforms is not suggestive of roles in cold stress responses, the recruitment to, and subsequent activity at gene promoters by BdGCN5 protein products may not be ruled out. Notably, *A. thaliana* GCN5 is known to interact with CBF1, a key transcription factor involved in the cold acclimation process (Stockinger et al., 2001). Furthermore, in both *A. thaliana* and *B. distachyon*, the SAGA complex member ADA2 has been shown to interact with CBF1 as well (Stockinger et al., 2001; Demone, 2012). As for high temperature exposure, the observed up-regulation of *BdGCN5* transcripts within the first hour of heat stress (Figure 3B) is consistent with observations made of *A. thaliana*'s *GCN5*, as well as of the role of *GCN5* in heat responses in *A. thaliana*, and more recently in *B. distachyon* (Hu et al., 2015; Martel, 2017).

ADA2 is a transcriptional co-activator that interacts with GCN5 in all organisms in which this interaction has been assessed, and is also a member of the SAGA complex (Candau and Berger, 1996; Candau et al., 1996, 1997; Stockinger et al., 2001; Bhat et al., 2003; Fan et al., 2004; Mao et al., 2006; Gamper et al., 2009). *A. thaliana* contains two copies of *ADA2* (*ADA2a* and *ADA2b*), however *B. distachyon* retains only one *ADA2* sequence in its genome, homologous to *A. thaliana*'s *ADA2a* (Stockinger et al., 2001; Demone, 2012). Therefore, a protein-protein interaction study was performed in *N. benthamiana* between *B. distachyon* ADA2, and BdGCN5 variants in order to determine whether each isoform is capable of multi-protein complex formation. Understanding this interaction is important as incorporation into such complexes would allow for the indirect recruitment of the histone acetyltransferase to additional genomic loci and the expansion of its catalytic capabilities by other SAGA-complex subunits (Belotserkovskaya et al., 2000; Kuo et al., 2000; Brown et al., 2001; Lang et al., 2001; Stockinger et al., 2001; Barbaric et al., 2003; Bhat et al., 2004; Gao et al., 2007; Nagy and Tora, 2007; Bian et al., 2011; Schram et al., 2013; Hirsch et al., 2015; Setiaputra et al., 2015). Exclusively GCN5 and L-GCN5 were capable of interacting with ADA2, indicating that these two isoforms would gain the increased function resulting from the SAGA complex association, whereas S-GCN5 would not (Figure 5). However, the low accumulation levels of the *S-GCN5* transcript (Figure 1D), in conjunction with the observation that it is degraded by NMD (Figures 2B,C), puts into question whether any S-GCN5 protein product accumulates in *B. distachyon* tissues. Previous examples are known to this effect, where splice variants that lose important catalytic domains do not display detectable protein accumulation levels (Schneider et al., 2005). Conversely, there are known examples of enzymes losing catalytic activity, including an acetyltransferase, through alternative splicing patterns, many of which are believed to modulate the level of active protein present in the cell (Kim et al., 2005; Schneider et al., 2005; Kelemen et al., 2013). However, while there is a single example that demonstrates that an NMD targeted transcript may be stabilized in order to be translated into protein, the predominant consensus is that transcripts targeted by NMD may not produce significant levels of protein product (Lareau et al., 2007; Saltzman et al., 2008; Colak et al., 2013; Kwon et al., 2014). Therefore, there are three possible fates for the *S*-GCN5 transcript: protein product may accumulate and function in the plant, this splice variant's role may be limited to transcriptional regulation, or it may simply be an aberrant transcript for which there is no significant function.

The confirmed interaction between GCN5/L-GCN5 and ADA2, but not between S-GCN5 and ADA2 may shed light upon the differences in higher-level protein organization of plant GCN5-containing complexes, when compared to those of other eukaryotic organisms. In yeast, the region required for the GCN5—ADA2 interaction is that present between the HAT and bromodomain (Candau and Berger, 1996; Candau et al., 1997). This region is missing in L-GCN5, which indicates that the interaction requirement is different in *B. distachyon* than in yeast. Furthermore, GCN5—ADA2 interaction studies performed with *A. thaliana* homologs (both ADA2a and ADA2b were assessed) indicated that this region was not required for protein-protein interaction (Mao et al., 2006). This *A. thaliana* study also demonstrated that the HAT domain was sufficient for GCN5—ADA2(a/b) interaction, which corroborates the *B. distachyon* interaction results where L-GCN5 associates with ADA2, while S-GCN5 does not (Figure 5; Mao et al., 2006). Taken together, these results suggest that the region necessary for the GCN5-ADA2 interaction in plants, and therefore its conformation, is different from that of yeast.

The above interaction study suggests that the *B. distachyon* SAGA complex could contain GCN5, L-GCN5, or a combination thereof in its HAT module. Furthermore, the proportion of GCN5-containing complexes would outnumber the L-GCN5 containing complexes if the absolute mRNA quantification data accurately correlates to protein accumulation levels. Therefore, *B. distachyon* most likely has alternative compositions of the SAGA complex that differ based on, at least, the BdGCN5 variant it harbors in its HAT module. These related complexes may differ significantly in their enzymatic capability and their preferred order of lysine acetylation, as was observed when a non-functional bromodomain was generated in yeast GCN5 (Cieniewicz et al., 2014). In addition, the loss of the bromodomain may significantly alter the sites at which L-GCN5-containing complexes may be recruited to, as was observed when the bromodomain was removed from GCN5 of yeast and *A. thaliana* (Hassan et al., 2002; Benhamed et al., 2008). If any S-GCN5 protein accumulates to significant levels in *B. distachyon*, its role would not gain functionality through interaction with the SAGA complex, which would limit its enzymatic capability and substrate specificity. In addition, as the HAT motifs S-GCN5 is missing are those that are most highly conserved and contain catalytic residues, an enzymatic function would be unlikely. However, NMD results indicate that S-GCN5 protein accumulation is unlikely, suggestive of a transcriptional role or *S-GCN5* being an aberrant splicing product. Therefore, these findings provide insight regarding the diversification of different BdGCN5 isoforms, a subset of which may act as a part of SAGA-like complexes. Further investigation is required to understand the functional differences that may be displayed among SAGA complexes containing different BdGCN5 isoforms.

## Author contributions

AM and J-BC: designed all experiments. HB: identified the alternative transcripts. AM: performed all experiments and analyses. BM: contributed to the BiFC experiment. AM, BM, and J-BC: prepared the manuscript.

### Conflict of interest statement

The authors declare that the research was conducted in the absence of any commercial or financial relationships that could be construed as a potential conflict of interest.

## Supplementary material

Supplementary Table 1 Primers used in this study.

Supplementary Figure 1 *BdGcn5* nucleotide and amino acid sequence alignment.

Supplementary Figure 2 Standard curves for absolute RNA quantification.

Supplementary Figure 3 *B. distachyon* nonsense-mediated decay gene homologs.
