# Supplementary figures and images for: Diversification of the Histone Acetyltransferase GCN5 through Alternative Splicing in Brachypodium distachyon
Source: Front Plant Sci. 2017 Dec 21;8:2176. doi: 10.3389/fpls.2017.02176 (PMC5743026; doi:10.3389/fpls.2017.02176)

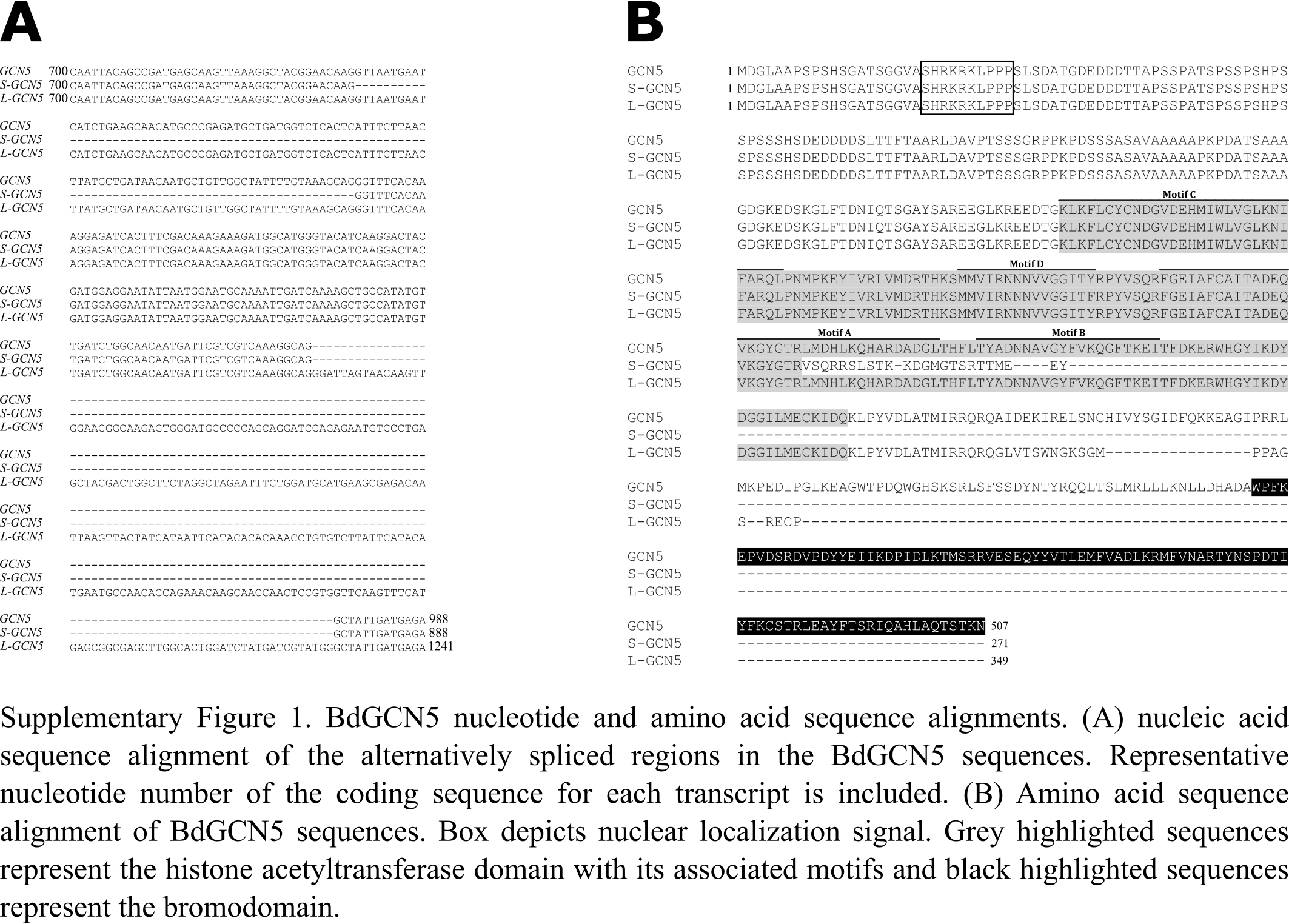

Supplement: Supplementary Figure 1 — BdGcn5 nucleotide and amino acid sequence alignment. [file Image1.JPEG]

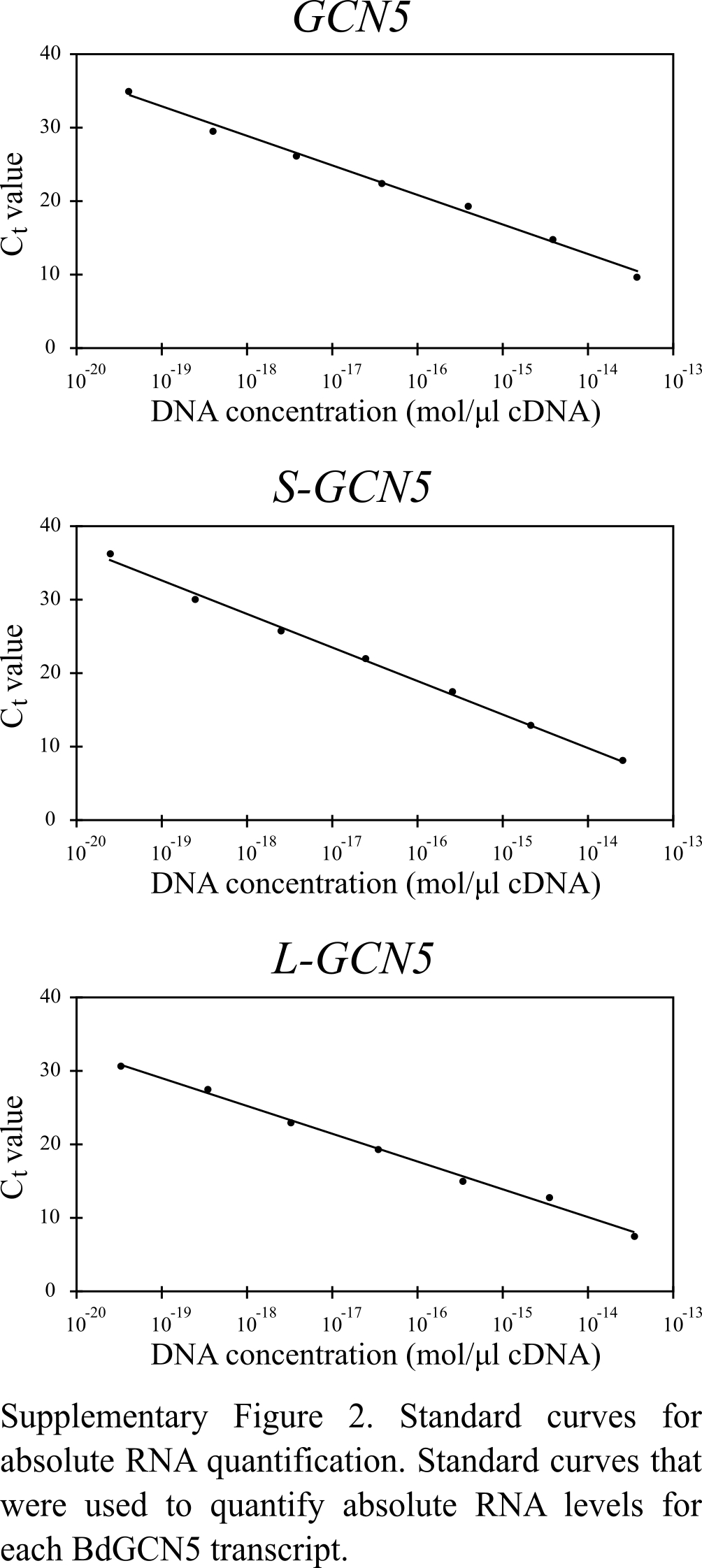

Supplement: Supplementary Figure 2 — Standard curves for absolute RNA quantification. [file Image2.JPEG]

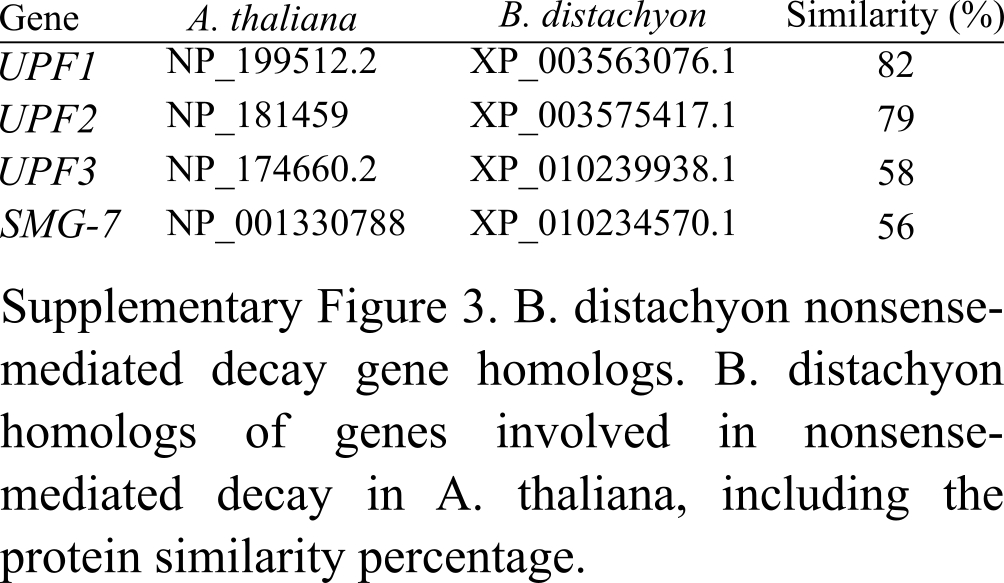

Supplement: Supplementary Figure 3 — B. distachyon nonsense-mediated decay gene homologs. [file Image3.JPEG]
